# Response to letter by Drs. Bottinger and van der Hoorn

**DOI:** 10.1186/s40635-019-0258-x

**Published:** 2019-06-06

**Authors:** David A. Berlin, Seth Manoach, Paul M. Heerdt

**Affiliations:** 1 Division of Pulmonary and Critical Care Medicine, Weill Cornell Medicine, 1300 York Avenue, New York, NY 10065 USA; 2 Division of Applied Hemodnamics, Yale University School of Medicine, New Haven, USA

We thank Drs. Bottinger and van der Hoorn for their close reading of our manuscript and their comments. As they point out, our study was designed to primarily evaluate how the relatively unique capability of expiratory ventilatory assistance (EVA) to generate negative end-expiratory pressure (NEEP) affected blood pressure and cardiac output flow in the setting of profound hemorrhage. For the study, after removing 40 mL/kg of blood to induce marked hypotension, subjects received either conventional positive pressure ventilation with 0 end-expiratory pressure or EVA ventilation with NEEP. Minute ventilation was matched for both groups and under these conditions, arterial CO2 was higher in the group receiving EVA ventilation. This difference needs to be interpreted in the context of two very important considerations.

First, the study was designed to assess potential hemodynamic benefits of EVA with NEEP not necessarily optimize ventilation. With NEEP of − 8 cmH2O, EVA required a peak inspiratory pressure of only ~ 6–7 cmH20 to produce a tidal volume and PaO2 similar to that generated by conventional ventilation with 0 end-expiratory pressure and a peak inspiratory pressure of 20 cmH20. The ability to ventilate with this low PIP is remarkable and clearly contributed to the observed hemodynamic benefit. It also suggests that during hemorrhagic shock, increased gas flow and higher PIP can be used at least intermittently in conjunction with NEEP without major loss of hemodynamic benefit. Ultimately, we agree with Drs. Bottinger and van der Hoorn that broad interpretation of our results in terms of CO2 removal is not warranted given the specific use of EVA with NEEP and conditions of our experimental protocol. We also agree that the experimental model is not indicative of the established clinical use of EVA rescue ventilation with an obstructed airway and that choice of the catheter and mode of ventilation could have affected the observed efficiency of gas exchange.

Second, study results indicate that the improvements in blood pressure and flow, in conjunction with maintenance of oxygenation, during EVA with NEEP outweigh any decrement in CO2 elimination with a fixed minute ventilation. In the absence of fluid administration or other resuscitative measures, 1 h after marked hemorrhage mean arterial pressure was double that in subjects with conventional ventilation and cardiac output nearly 60% higher. This relatively profound response may have particular short-term benefit in a clinical setting where it is highly likely that volume resuscitation will be implemented sooner and ventilation can be adjusted based upon blood gas analysis.

## Data Availability

All raw data will be made available in the appendix

